# Generative AI Use, Perceived Usefulness, Perceived Risk, and Physician Burnout and Fulfillment Among Chinese Physicians: Mixed Methods Multiregional Study

**DOI:** 10.2196/94155

**Published:** 2026-09-11

**Authors:** Dan Guo, Yanan Zhao, Tingkun Yang, Xingyu Bao, Ping Huang

**Affiliations:** 1 Department of Scientific Research and Teaching Beijing Genertec Aerospace Hospital Beijing China; 2 Faculty of Health and Wellness City University of Macau Macau China; 3 Department of Child Health Care Luzhou People’s Hospital Chengdu China

**Keywords:** generative artificial intelligence, perceived usefulness, perceived risk, burnout, professional fulfillment

## Abstract

**Background:**

As generative AI (GenAI) becomes increasingly prevalent, its impact on physician mental health has garnered significant attention; yet, empirical evidence remains limited.

**Objective:**

This study aims to investigate the correlations between the usage frequency of GenAI, perceived usefulness (PU), and perceived risk (PR) of GenAI with physicians’ burnout and professional fulfillment.

**Methods:**

A mixed methods design was used, integrating a quantitative survey of physicians across 4 regions in China with in-depth qualitative interviews to elucidate the underlying psychological mechanisms. The quantitative component involved a cross-sectional survey of 961 physicians, with the questionnaire collecting data on demographic and professional characteristics, socioeconomic status, GenAI usage frequency, PU, and PR. Semistructured interviews with 10 physicians were used for in-depth mining. Multivariable logistic and linear regression models with province-level fixed effects were fitted to examine the association between usage of GenAI, PU, PR, and physicians’ burnout and fulfillment. Stratified analyses were further performed to explore the moderating effect of demographic and clinical characteristics.

**Results:**

Quantitative analysis revealed no direct correlation between GenAI usage frequency and burnout. However, PU was positively associated with professional fulfillment (odds ratio [OR] 1.56, 95% CI 1.17-2.08; *P*=.003), whereas PR was associated with a higher likelihood of burnout (OR 1.80, 95% CI 1.46-2.21; *P*<.001). Stratified analyses showed that for physicians working ≥3 night shifts per week, GenAI usage was associated with higher odds of burnout, although the estimate was imprecise (OR 13.96, 95% CI 2.40-81.04; *P*=.003). The qualitative findings further suggested that the benefits of using GenAI may be offset by the additional burden. The PU of GenAI was perceived to enhance professional fulfillment by bolstering self-efficacy, whereas the PR of GenAI was linked to heightened burnout rooted in unclear boundaries of responsibilities and rights, as well as challenges to professional identity.

**Conclusions:**

The GenAI revolution in medicine is as much a psychological transition as it is a technological one. GenAI use is not directly associated with improved psychological states among clinicians. The PU of GenAI relates to professional fulfillment, and the PR concerns correspond to elevated burnout. Sustaining clinician well-being during this digital shift thus parallels a dual requirement, balancing the potential for professional fulfillment tied to GenAI utility against the concurrent verification fatigue and legal uncertainty cluster around clinician burnout.

## Introduction

Physician burnout has emerged as a global public health crisis [1], characterized by work exhaustion, interpersonal disengagement, and a diminished sense of professional accomplishment [2]. The 2018 Survey of America’s Physicians Practice Patterns and Perspectives reported that 77.8% of physicians experienced burnout [3]. In China’s rapidly evolving health care system, clinicians face intensified workloads and escalating clinical demands. A large-scale, cross-sectional study conducted across 6 representative provinces in China revealed that the prevalence of professional burnout among Chinese physicians reached 60.8% [4]. Physician burnout is associated with compromised patient safety [5], increased risk of medical errors [6,7], and significant secondary psychological distress, including depression and suicidal ideation [8].

The rapid integration of generative AI (GenAI) is fundamentally reshaping clinical practice [9]. From automating medical documentation to providing sophisticated research assistance and diagnostic support, GenAI is heralded as a transformative solution to liberate physicians from escalating administrative burdens [10]. However, a paradoxical reality remains: despite these technological advancements, global rates of physician burnout remain at historic highs. While GenAI promises to liberate physicians from mundane tasks [11,12], its impact on their psychological health remains a subject of intense debate. The relationship between GenAI’s potential for efficiency and its actual impact on health care providers has become a critical area of inquiry [13].

While several studies suggest that GenAI can alleviate administrative burdens and mitigate clinician burnout, existing findings remain inconsistent. A multicenter quality improvement study in the United States demonstrated that the implementation of a GenAI-enabled documentation assistant was associated with a significant reduction in clinician burnout, which decreased from 51.9% to 38.8% (odds ratio [OR] 0.26, 95% CI 0.13-0.54). Furthermore, significant improvements were observed in secondary outcomes, including documentation-related cognitive load, after-hours charting time, and patient engagement [14]. One review investigated the impact of GenAI (large language models) on physician burnout within the context of medical documentation and administrative tasks. It indicated that GenAI integration could mitigate burnout by reducing documentation time, enhancing the quality of physician-patient interactions, and optimizing clinical workflows, with burnout prevalence dropping from 69% to 43% [15]. A systematic review of 13 studies found that although GenAI-enabled scribes are promoted as tools to alleviate clinical documentation burdens and mitigate physician burnout, current evidence remains limited. The study highlighted that existing research has yet to fully substantiate the capacity of these technologies to yield meaningful time savings or a significant reduction in cognitive load [16]. A cross-sectional survey involving 6726 Chinese radiologists revealed that, compared to those not using GenAI, the use of GenAI was significantly associated with an increased risk of professional burnout (OR 1.20, 95% CI 1.10-1.30) [17]. However, another study indicated that radiologists who spent more time using GenAI tended to experience less burnout [18]. In addition, emerging yet limited research suggests that physicians’ psychological perceptions of GenAI significantly influence their level of burnout or well-being. For instance, a study conducted at a West Coast hospital in the United States demonstrated that positive perceptions of GenAI, specifically GenAI trust, are associated with a reduction in individual-level burnout [19]. Similarly, a cross-sectional survey of physicians in public hospitals in the Kingdom of Saudi Arabia revealed that health care professionals with more favorable perceptions of GenAI reported better psychological well-being [20].

Beyond these inconsistent findings, the existing literature also leaves a critical gap in understanding the association between physicians’ perceptions of GenAI and the dual outcomes of burnout and professional fulfillment in the context of China’s rapidly digitizing health care system. Based on the job demands-resources (JD-R) theory [21] and the technology acceptance model (TAM) [22-24] frameworks, this study uses a mixed methods design, combining a large-scale multiregional survey with in-depth interviews to capture both the statistical trends and the experiences and psychological feelings of physicians. While perceived usefulness (PU) and perceived risk (PR) originate from TAM, traditionally focused on adoption behaviors, within modern clinical workflows, these perceptions function as environmental factors rather than mere adoption precursors. Specifically, PU is operationalized as a cognitive job resource—an instrumental multiplier. Conversely, PR is operationalized as a psychological job demand, whereby heightened risk perceptions track with constant hyper-vigilance and cognitive friction. This theoretical translation aligns technology-specific appraisals with clinician well-being outcomes within the JD-R matrix. Therefore, this study aims to examine the associations between the usage frequency of GenAI, PU, and PR of GenAI and physician well-being, providing empirical evidence that highlights the potential value of optimizing digital health tools to mitigate occupational burnout.

## Methods

This study adopted a sequential explanatory mixed methods design, wherein the qualitative phase directly followed and built upon the preliminary quantitative analysis. We first conducted a quantitative analysis to examine cross-sectional associations between GenAI usage, perceptions, and physician occupational outcomes. Subsequently, a qualitative exploration was deployed to uncover the underlying mechanisms and contextual nuances explaining these quantitative findings.

### Part I: Quantitative Analysis

#### Study Design and Sampling

This study used a cross-sectional survey design with multistage purposive sampling to ensure contextual diversity and theoretical relevance. Four geographically diverse regions in China were selected based on institutional access feasibility to maximize variation across two key dimensions: (1) economic development level and (2) geographic location. The selected sites comprised Fengtai-Beijing (gross domestic product [GDP] per capita: CNY ¥200,278 [CNY ¥1=US $0.14 as of January 26, 2026] in 2023, representing 2.2 times the national average), representing a highly developed metropolitan area with a dense concentration of medical resources; Taiyuan-Shanxi Province (GDP per capita: ¥102,922, 1.1 times the national average), representing a central Chinese provincial capital with a transitioning industrial economy; Luzhou-Sichuan Province (GDP per capita: ¥63,913 in 2023, approximately equivalent to 72% of the national average), representing southwestern developing regional health care contexts; and Ningde-Fujian Province (GDP per capita: ¥120,619, 1.3 times the national average), representing a southeastern coastal city experiencing rapid economic growth. This multiregional approach balanced practical feasibility with the imperative to capture diversity in physicians’ professional contexts, thereby enhancing the transferability of findings beyond single geographic settings. Economic development data were obtained from the National Bureau of Statistics of China [25].

#### Sampling Strategy

Within each region, we recruited physicians from hospitals of different tiers (tertiary and secondary). We used purposive sampling, aiming to maximize variation in professional seniority, department type, and work conditions. Inclusion criteria required active clinical practice as a licensed physician at the time of the survey. Participant recruitment occurred from September to November 2025. We approached 1142 physicians from tertiary and secondary hospitals across 4 regions. After excluding 181 physicians with incomplete survey responses or those from ineligible departments or regions, the final analytical sample comprised 961 physicians (see Figure S1 in Multimedia Appendix 1). Regional distribution was as follows: Fujian (n=619, 64.4%), Sichuan (n=127, 13.2%), Beijing (n=118, 12.3%), and Shanxi (n=97, 10.1%). While sample distribution reflects differential institutional access and recruitment feasibility rather than population proportions, our primary research focus is on individual-level factors (professional seniority, work conditions, and psychological well-being) rather than regional comparisons. Table S1 in Multimedia Appendix 1 provides detailed regional characteristics. The target sample size was calculated a priori using G*Power 3.1. For binary logistic regression with 15 predictors, detecting a small-to-medium effect size (OR ≈ 1.8) with 80% power and α=0.05 required a minimum of 800 respondents. Anticipating a 20% invalid response rate, we targeted 960-1000 participants, achieving a final sample of 961 complete responses with adequate power for all planned analyses.

#### Measures

##### Physician Professional Fulfillment and Burnout

Professional fulfillment and burnout were based on the previously validated professional fulfillment index (PFI) [2]. The PFI includes 3 subscales: professional fulfillment (6 items), work exhaustion (4 items), and interpersonal disengagement (6 items). Each of the subscales is rated using a 5-point Likert scale from 0 to 4, with higher scores indicating higher levels of fulfillment, exhaustion, and disengagement. The scores are then averaged for the subscales. Work exhaustion and interpersonal disengagement can be averaged together as an overall measure of burnout. A cutoff score of 3.0 is recommended for professional fulfillment, and a cutoff of 1.33 is used for burnout [2,26].

##### GenAI Usage, PU, and PR

GenAI usage was assessed by asking respondents: “In your work, do you use GenAI tools?” Response options were “mostly,” “occasionally,” “rarely,” and “never.” Both GenAI PU and PR were measured using 3-item scales adapted from established literature [22,23]. Participants were asked to consider the actual or anticipated impact of GenAI on their practice across 3 dimensions of PU: improved diagnostic accuracy, enhanced documentation efficiency, and overall task efficiency. Each item was rated on a 5-point Likert scale, ranging from 1 (strongly disagree) to 5 (strongly agree). The final PU score was calculated as the mean of the 3 items, with higher scores reflecting a more positive perception of GenAI utility. PR was similarly assessed using a 3-item scale focusing on the potential negative implications of GenAI in health care. The items addressed concerns regarding increased risk of clinical harm, exacerbation of health care inequality, and excessive patient reliance on GenAI in lieu of professional medical intervention. Agreement was rated on a 5-point Likert scale (1=strongly disagree, 5=strongly agree). The final PR score was calculated as the mean of the items, where higher scores indicate greater perceived risk of GenAI. Detailed survey items for both PU and PR are provided in Table S1 in Multimedia Appendix 2.

##### Validity and Reliability

To ensure the robustness of the measurement instrument, we assessed the reliability and validity of the perceived GenAI characteristics scales (PU and PR). Internal consistency was evaluated using Cronbach α and composite reliability (CR), with values exceeding 0.70 for both indicators, widely recognized as demonstrating acceptable reliability. Convergent validity (CV) was assessed via standardized factor loadings and average variance extracted (AVE). CV is established when standardized loadings are greater than 0.5, and the AVE exceeds the recommended threshold of 0.5. As demonstrated in Table S2 in Multimedia Appendix 2, all items demonstrated high factor loadings, and both the α and CR values for PU and PR surpassed the 0.8 threshold. The AVE values (AVE_PU_=0.59, AVE_PR_=0.63) further confirmed satisfactory CV for all constructs. Regarding discriminant validity, evaluation was conducted using the Fornell-Larcker criterion (Table S3 in Multimedia Appendix 2). The square roots of the AVE for PU (0.767) and PR (0.795) each exceeded the absolute value of the correlation between the two constructs (*r*=–0.030), confirming that PU and PR are statistically distinct dimensions. The initial pool of items was developed based on an extensive literature review and adapted from validated scales. To ensure content validity, the items were reviewed by 2 experts in public health to verify their relevance to the medical GenAI context. Prior to the formal survey, the questionnaire was pilot-tested with a small group of physicians to ensure the clarity of language and ease of understanding.

The PFI demonstrated reliability within our sample. Cronbach α coefficients were 0.92 for professional fulfillment, 0.92 for work exhaustion, and 0.94 for interpersonal disengagement. The combined scale for overall work burnout also displayed robust internal consistency (α=0.95). Confirmatory factor analysis (CFA) further indicated acceptable construct validity, confirming the hypothesized 3D structure with an overall acceptable model fit: comparative fit index (CFI)=0.93, Tucker-Lewis Index (TLI)=0.91, root-mean-square error of approximation (RMSEA)=0.10, and standardized root-mean-square residual (SRMR)=0.04. Although the RMSEA was slightly elevated at 0.10, the incremental fit indices (CFI and TLI) both clearly exceeded the 0.90 benchmark, and the SRMR indicated an excellent residual fit at 0.04. Collectively, these parameters substantiated robust structural validity for the measurement framework.

##### Covariate

The covariates included in this study were age, gender, marital status, ethnicity, salary satisfaction, professional title, years of practice, night-shift status, weekly patient volume, specialty, teaching, and research assessment pressure. All variables were obtained via self-reporting by the participants. Age was categorized into three groups: ≤35 years, 36-45 years, and ≥46 years. Salary satisfaction was rated on a 5-point scale from 1 (very dissatisfied) to 5 (very satisfied), with higher scores indicating greater satisfaction. Physicians’ years of practice were categorized into five groups: 1-5, 6-10, 11-15, 16-20, and ≥21 years. Night-shift status was classified based on weekly frequency: ≥3 nights per week, 1-2 nights per week, and no night shifts. Weekly patient volume was grouped into three levels: 1-20 patients, 21-50 patients, and ≥51 patients. Based on a theoretical framework of professional paradigms and GenAI applicability, specialty is categorized into four types: (1) diagnostic and analytical type (DAT, eg, radiology), whose core work relies on the interpretation of objective data; (2) procedural and operative type (POT, eg, surgery), which emphasizes manual skills and procedural operations; (3) decision-making type (DMT, eg, internal medicine), which focuses on evidence-based decision-making and long-term patient relationships; and (4) relational and communicative type (RCT, eg, psychiatry), which is centered around communication and subjective assessment. This classification draws upon prior research concerning the differential integration of GenAI across various medical fields [27,28]. Participants were first surveyed on whether they were subject to teaching performance requirements. For those responding affirmatively, a follow-up question measured the perceived pressure of fulfilling these requirements. Respondents reporting relatively high or very high pressure were categorized into the high-pressure group. In contrast, participants without teaching performance requirements, as well as those with such requirements but perceiving no pressure, a little pressure, or moderate pressure, were classified into the “no or low teaching pressure” group. The same measurement and classification approach was applied to research assessment pressure.

#### Statistical Analysis

Frequencies and percentages were used to describe the sociodemographic, professional characteristics, and GenAI usage patterns of the participants. Differences between groups were assessed using the chi-square test, *t* test, or ANOVA. To address potential regional heterogeneity, we used multivariable logistic and linear regression models with province-level fixed effects. Given that our data originated from 4 distinct provinces, we included province dummy variables to control for unobserved province-specific factors, such as regional health care infrastructure and local policy variations. This approach allowed us to focus on the individual-level associations between GenAI use, PU and PR of GenAI with burnout, fulfillment, and PFI dimension scores, adjusting for basic demographic information, socioeconomic status, and workload. GenAI use was analyzed as a categorical variable to illustrate the association. For the main analysis, GenAI usage was divided into 4 groups: mostly, occasionally, rarely, and never. For the stratified analyses, to avoid low cell counts and ensure robust estimations, the exposure was dichotomized: mostly, occasionally, and rarely were collapsed into a single GenAI user group, while never was designated as the nonuser reference group. The years in practice were also dichotomized into two groups (1-10 years and ≥11 years) in the stratified analyses. This binary classification was adopted for two reasons: first, to ensure adequate statistical power and sample size within each subgroup during stratification; second, to meaningfully distinguish junior physicians from senior physicians. Burnout and professional fulfillment derived from the PFI were incorporated into the model as binary variables, with cutoff values of 1.33 and 3, respectively, as stated in the measurement section. Meanwhile, the two dimensions of burnout, including work exhaustion and interpersonal disengagement, were entered into the model as continuous variables based on their averaged scores. We analyzed the correlations between the independent variables related to GenAI and burnout, as well as fulfillment, and also examined the relationships between the independent variables and the scores of each dimension of burnout. We also conducted stratified analyses to verify the heterogeneity of these correlations across subgroups. To ensure the stability and reliability of the models, we conducted a multicollinearity assessment by calculating the variance inflation factors (VIFs) for all included covariates. The results indicated that the VIF values for all individual variables ranged from 1.06 to 4.56, which were well below the conservative threshold of 5.0. The overall mean VIF of the model was 2.18. To assess common method variance (CMV), we conducted Harman’s single-factor test using an unrotated exploratory factor analysis (EFA). All perceptual items (PU, PR, GenAI usage, burnout, and fulfillment) were entered into the analysis. The results showed that the first factor accounted for 35.4% of the total variance. Since this value is well below the 50% threshold, CMV is not considered a significant threat to the validity of our findings. While the first factor captured 35.4% of the variance, the remaining 64.6% was distributed across other factors, further confirming the multidimensional structure of our measurement model. Results were presented as ORs or regression coefficients (β) with 95% CIs. The significance level was set at *P*<.05. All statistical analyses were conducted in Stata 16.0 (StataCorp LP).

### Part II: Qualitative Analysis

#### Study Design and Sampling Strategy

To further elucidate the mechanisms underlying the quantitative findings, we conducted a qualitative substudy using semistructured interviews. The interview guide was developed through a sequential explanatory design [29], where specific prompts were tailored to probe the counterintuitive findings from the quantitative phase, such as the efficiency paradox—the null association between GenAI use and burnout. We sought to explore the deep-seated reasons why physicians’ perceptions (PU and PR) of GenAI differentially impact their burnout or well-being. The interview content included the current level of burnout and its specific manifestations, the scenarios where GenAI is used, the impact of GenAI usage on work, the positive feedback mechanism of GenAI PU on job fulfillment or satisfaction, specific sources of anxiety and scenarios behind the PR of GenAI, and the difficulties and demands encountered during GenAI usage.

A combination of purposive and maximum variation sampling was used to recruit participants across diverse clinical specialties, professional experience levels, and burnout profiles until thematic saturation was achieved (N=10, see Table S1 in Multimedia Appendix 3). Since the quantitative survey was anonymous, the qualitative interviewees were recruited independently. To unpack discordant and heterogeneous quantitative findings, we enriched our maximum variation sample by intentionally recruiting participants who manifested specific profiles mirroring our quantitative anomalies, such as clinicians regularly undergoing high-intensity night shifts and high-frequency GenAI users experiencing work exhaustion. Following empirical benchmarks for homogeneous professional cohorts [30], we defined thematic saturation as the point at which no new subthemes, codes, or conceptual dimensions emerged from the data. Codebook stability was evaluated dynamically; foundational themes had fully emerged by the fifth interview, and the final 5 interviews yielded no new unique information, confirming that thematic saturation had been reached (the thematic saturation matrix is provided in Table S2 in Multimedia Appendix 3). Data collection took place in December 2025 via a combination of face-to-face interviews and Tencent Meeting.

#### Qualitative Rigor and Trustworthiness

Transcripts were analyzed following Braun and Clarke’s 6-step thematic analysis framework [31]. To ensure methodological rigor, several quality indicators were systematically addressed.

##### Reflexivity

We openly disclose that the lead author is clinically active within one of the primary hospital systems included in this study. While this “insider status” facilitated deep contextual rapport and allowed for highly candid, jargon-rich accounts from peer physicians, strict measures were implemented to mitigate confirmation bias. All audio recordings were thoroughly deidentified and pseudonymized (participants 1-10) prior to analysis; two researchers (DG and TY) coded the data independently, and discrepancies were resolved by consensus. To guard against overinterpretation or selective attention to confirmatory evidence, two coauthors (YZ and XB) independently reviewed the coding framework.

##### Coding Reliability

During the verbatim transcription of digital recordings, we immersed ourselves in the data and identified recurring keywords such as “responsibility,” “efficiency enhancement,” and “exhaustion.” We then generated initial codes by labeling meaningful segments. To ensure coding reliability, two researchers coded the data independently. The initial interrater percentage agreement was 83.5%, demonstrating high baseline coding consistency. Any remaining discrepant code assignments were subsequently resolved through iterative reflexivity meetings until a 100% consensus on the final thematic hierarchy was achieved. Related codes were subsequently clustered to form preliminary themes.

##### Thematic Verification and Respondent Validation

These themes were iteratively verified against the raw data to ensure contextual consistency. Furthermore, postanalysis member checking was conducted by sharing descriptive summaries of the generated themes with 3 available participants, who formally validated that the conceptual structure accurately reflected their lived professional realities. The essence of each theme was refined and articulated using formal academic terminology. Finally, these consolidated themes constituted the qualitative findings of this study.

### Ethical Considerations

The study received ethics approval from the Institutional Review Board of City University of Macau (approval no. FHW-ER-2526-004). Participation was voluntary with electronic informed consent. Participants were not compensated for their participation. Data were collected anonymously and stored securely in accordance with institutional data protection policies. All materials have been deidentified to ensure that no individual participant or user can be identified.

## Results

### Participants’ Personal and Professional Characteristics

Table 1 shows the demographic and professional characteristics of the participating physicians. Among the 961 participants, the frequency of GenAI use was as follows: 101 physicians (10.50%) reported mostly using GenAI, 485 (50.47%) occasionally used it, 216 (22.48%) rarely used it, and 159 (16.55%) never used GenAI. The majority of the participants were young physicians, with those under 35 years old accounting for 51.51%. Women made up 51.72%, and married participants accounted for 67.22%. As shown in Table 1, physicians aged 36-45 years, with 16-20 years in practice, no night-shift duties, specializing in DAT, working in Shanxi Province, and who were under high research assessment pressure exhibited the highest frequency of GenAI use (*P*<.05).

**Table 1:** Personal and professional characteristics of the study population: a cross-sectional survey of physicians across multiple provinces in China (2025, N=961).

Characteristics		Total (N=961)	The frequencies of using generative AI					Chi-square (*df*)/*F* test (*df*)		*P* value
			Mostly (n=101)	Occasionally (n=485)	Rarely (n=216)	Never (n=159)				
**Age (years), n (%)**							12.96^a^ (6)		.04	
	≤35	495 (51.51)	49 (9.90)	236 (47.68)	122 (24.65)	88 (17.78)				
	36-45	310 (32.26)	38 (12.26)	172 (55.48)	63 (20.32)	37 (11.94)				
	≥46	156 (16.23)	14 (8.97)	77 (49.36)	31 (19.87)	34 (21.79)				
**Gender, n (%)**							1.37^a^ (3)		.71	
	Male	464 (48.28)	49 (10.56)	238 (51.29)	97 (20.91)	80 (17.24)				
	Female	497 (51.72)	52 (10.46)	247 (49.70)	119 (23.94)	79 (15.90)				
**Marital status, n (%)**							6.92^a^ (3)		.07	
	Married	646 (67.22)	60 (9.29)	326 (50.46)	158 (24.46)	102 (15.79)				
	Other	315 (32.78)	41 (13.02)	159 (50.48)	58 (18.41)	57 (18.10)				
**Ethnicity, n (%)**							1.56^a^ (3)		.66	
	Han	924 (96.15)	98 (10.61)	469 (50.76)	206 (22.29)	151 (16.34)				
	Non-Han	37 (3.85)	3 (8.11)	16 (43.24)	10 (27.03)	8 (21.62)				
Salary satisfaction, mean (SD)		3.11 (1.13)	3.05 (1.28)	3.09 (1.11)	3.16 (1.09)	3.13 (1.18)	0.31^b^ (3, 957)		.82	
**Professional title, n (%)**							15.70^a^ (9)		.07	
	Primary	278 (28.93)	35 (12.59)	139 (50.00)	56 (20.14)	48 (17.27)				
	Intermediate	253 (26.33)	20 (7.91)	133 (52.57)	66 (26.09)	34 (13.44)				
	Senior	283 (29.45)	34 (12.01)	149 (52.65)	58 (20.49)	42 (14.84)				
	Other	147 (15.30)	12 (8.16)	64 (43.54)	36 (24.49)	35 (23.81)				
**Years in practice, n (%)**							34.16^a^ (12)		.001	
	1-5	297 (30.91)	39 (13.13)	138 (46.46)	63 (21.21)	57 (19.19)				
	6-10	200 (20.81)	11 (5.50)	105 (52.50)	55 (27.50)	29 (14.50)				
	11-15	177 (18.42)	15 (8.47)	100 (56.50)	43 (24.29)	19 (10.73)				
	16-20	119 (12.38)	24 (20.17)	52 (43.70)	22 (18.49)	21 (17.65)				
	≥21	168 (17.48)	12 (7.14)	90 (53.57)	33 (19.64)	33 (19.64)				
**Night shifts per week, n (%)**							21.73^a^ (6)		.001	
	≥3 days	112 (11.65)	11 (9.82)	45 (40.18)	28 (25.00)	28 (25.00)				
	1-2 days	654 (68.05)	64 (9.79)	350 (53.52)	153 (23.39)	87 (13.30)				
	No	195 (20.29)	26 (13.33)	90 (46.15)	35 (17.95)	44 (22.56)				
**Weekly patient volume, n (%)**							10.16^a^ (6)		.12	
	1-20	338 (35.17)	37 (10.95)	175 (51.78)	66 (19.53)	60 (17.75)				
	21-50	265 (27.58)	17 (6.42)	136 (51.32)	68 (25.66)	44 (16.60)				
	≥51	358 (37.25)	47 (13.13)	174 (48.60)	82 (22.91)	55 (15.36)				
**Specialty, n (%)**							23.78^a^ (9)		.005	
	DAT^c^	132 (13.74)	27 (20.45)	60 (45.45)	23 (17.42)	22 (16.67)				
	POT^d^	321 (33.40)	24 (7.48)	181 (56.39)	70 (21.81)	46 (14.33)				
	DMT^e^	392 (40.79)	38 (9.69)	186 (47.45)	99 (25.26)	69 (17.60)				
	RCT^f^	116 (12.07)	12 (10.34)	58 (50.00)	24 (20.69)	22 (18.97)				
**Province, n (%)**							23.05^a^ (9)		.006	
	Fujian	619 (64.41)	51 (8.24)	312 (50.40)	154 (24.88)	102 (16.48)				
	Beijing	118 (12.28)	17 (14.41)	61 (51.69)	20 (16.95)	20 (16.95)				
	Sichuan	127 (13.22)	14 (11.02)	58 (45.67)	29 (22.83)	26 (20.47)				
	Shanxi	97 (10.09)	19 (19.59)	54 (55.67)	13 (13.40)	11 (11.34)				
**Teaching assessment pressure, n (%)**							5.78^a^ (3)		.12	
	No or low-pressure	713 (74.19)	70 (9.82)	350 (49.09)	166 (23.28)	127 (17.81)				
	High-pressure	248 (25.81)	31 (12.50)	135 (54.44)	50 (20.16)	32 (12.90)				
**Research assessment pressure, n (%)**							15.91^a^ (3)		.001	
	No or low-pressure	601 (62.54)	55 (9.15)	287 (47.75)	140 (23.29)	119 (19.80)				
	High-pressure	360 (37.46)	46 (12.78)	198 (55.00)	76 (21.11)	40 (11.11)				

^a^Chi-square values.

^b^*F* values.

^c^DAT: diagnostic and analytical type.

^d^POT: procedural and operative type.

^e^DMT: decision-making type.

^f^RCT: relational and communicative type.

### Differences in GenAI Use Frequency, PU, and PR Scores by Burnout and Fulfillment Status

Table 2 presents the descriptive comparison of GenAI usage and perceptions across different groups. There was no statistically significant difference in the frequency of GenAI usage between physicians with and without burnout (*P*=.37), nor between those with and without high professional fulfillment (*P*=.13). However, significant descriptive differences were observed in the perceptions of GenAI. Physicians without burnout reported higher PU scores compared to those with burnout (mean 3.38, SD 0.74, vs mean 3.27, SD 0.64; *P*=.02). Similarly, physicians with high professional fulfillment exhibited substantially higher PU scores than those without (mean 3.51, SD 0.77, vs mean 3.24, SD 0.63; *P*<.001). Physicians with burnout perceived higher risks than their nonburnout counterparts (mean 2.86, SD 0.74, vs mean 2.41, SD 0.86; *P*<.001). Those with high professional fulfillment reported lower PR scores (mean 2.58, SD 0.94, vs mean 2.75, SD 0.76; *P*=.004).

**Table 2:** Differences in generative AI (GenAI) usage frequency, perceived usefulness (PU), and perceived risk (PR) scores among Chinese physicians by burnout and high fulfillment status.

		Burnout				High fulfillment		
		No	Yes	*P* value	No		Yes	*P* value
**GenAI usage frequency**				.37				.13
	Mostly	40 (39.60)	61 (60.40)		67 (66.34)		34 (33.66)	
	Occasionally	150 (30.93)	335 (69.07)		376 (77.53)		109 (22.47)	
	Rarely	72 (33.33)	144 (66.67)		164 (75.93)		52 (24.07)	
	Never	55 (34.59)	104 (65.41)		120 (75.47)		39 (24.53)	
PU, mean (SD)		3.38 (0.74)	3.27 (0.64)	.02	3.24 (0.63)		3.51 (0.77)	<.001
PR, mean (SD)		2.41 (0.86)	2.86 (0.74)	<.001	2.75 (0.76)		2.58 (0.94)	.004

### PU and PR Item Scores Across Physician Burnout and Fulfillment Status

**Figure 1:**
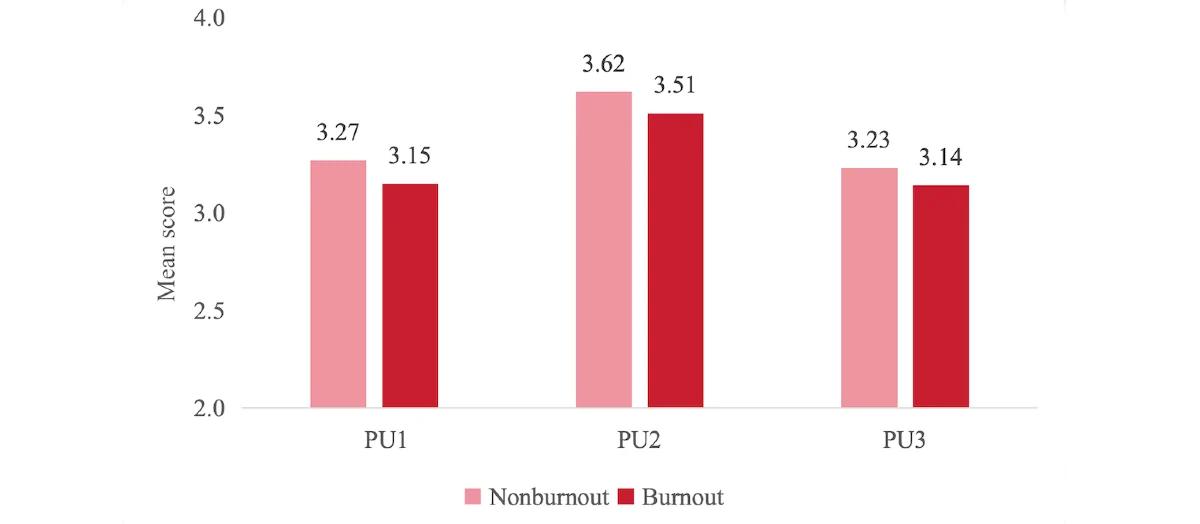
Mean perceived usefulness (PU) scores of generative AI (GenAI) by items and physician burnout status.

**Figure 2:**
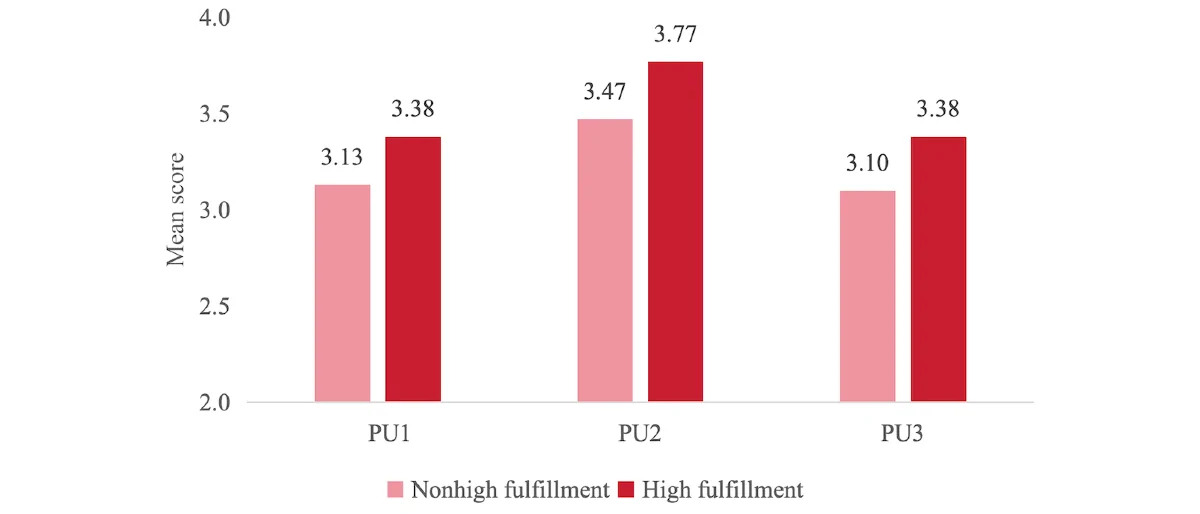
Mean perceived usefulness (PU) scores of generative AI (GenAI) by item and physician fulfillment status.

In the comparison between the burnout and nonburnout groups, mean scores for all three PU items (PU1, PU2, and PU3) remained relatively stable, with scores generally ranging between 3.14 and 3.62. Although the nonburnout group exhibited slightly higher mean scores across all items compared to the burnout group, the differences were modest, as shown in Figure 1. Conversely, physicians in the high professional fulfillment group reported higher scores on all 3 PU items than those in the nonhigh fulfillment group, as shown in Figure 2.

**Figure 3:**
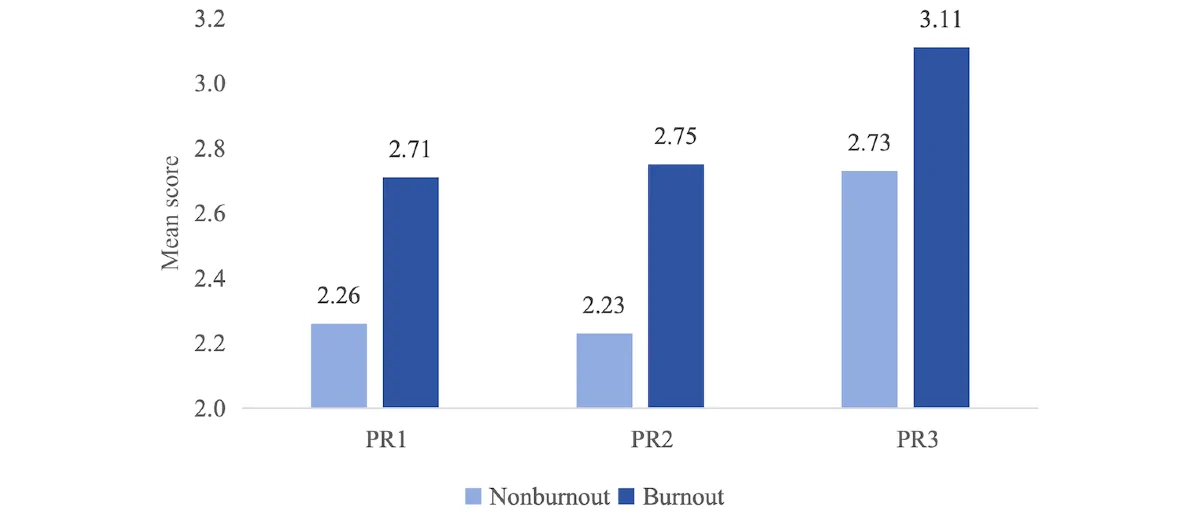
Mean perceived risk (PR) scores of generative AI (GenAI) by items and physician burnout status.

**Figure 4:**
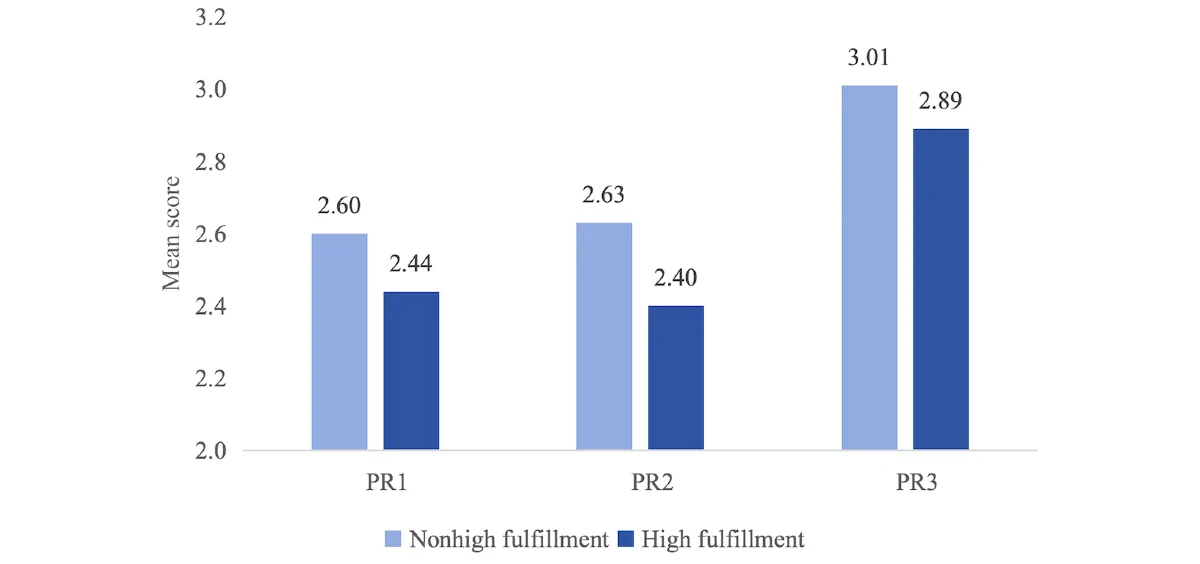
Mean perceived risk (PR) scores of generative AI (GenAI) by item and physician fulfillment status.

Figure 3 illustrates that physicians in the burnout group reported higher scores across all 3 PR items compared to those in the nonburnout group. Conversely, physicians with high professional fulfillment exhibited lower PR scores on all 3 items than their counterparts with low fulfillment, as shown in Figure 4.

### Associations of GenAI Usage, PU, and PR With Burnout and Fulfillment

Table 3 shows the associations of GenAI usage, PU, and PR with physician burnout and high fulfillment based on the fixed-effects logistic regression models. Overall, after fully adjusting for all potential covariates, both PU and PR remained significantly associated with burnout or high fulfillment. In the crude and adjusted models, compared with physicians who used GenAI “mostly,” those with lower frequencies of usage showed a higher likelihood of burnout and a lower likelihood of high fulfillment, though these associations were nonsignificant. In the crude models, higher PU was significantly linked to a lower risk of burnout (OR 0.79, 95% CI 0.65-0.97; *P*=.02) and a higher likelihood of high fulfillment (OR 1.85, 95% CI 1.48-2.33; *P*<.001). Conversely, higher PR was associated with higher odds of burnout (OR 2.09, 95% CI 1.74-2.51; *P*<.001) and lower fulfillment odds (OR 0.77, 95% CI 0.64-0.92; *P*=.005). In the fully adjusted models, perceptions of GenAI were strong independent factors associated with both outcomes. Specifically, higher PU scores were significantly associated with a higher likelihood of experiencing professional fulfillment (OR 1.56, 95% CI 1.17-2.08; *P*=.003). Meanwhile, PR retained an independent positive association with burnout (OR 1.80, 95% CI 1.46-2.21; *P*<.001).

**Table 3:** Associations of generative AI (GenAI) usage, perceived usefulness (PU), and perceived risk (PR) with physician burnout and high fulfillment: a fixed-effects logistic regression analysis.

				Burnout			High fulfillment	
				OR (95% CI)	*P* value	OR (95% CI)		*P* value
**Model^a^**								
	**The frequencies of using GenAI**							
		Mostly	Reference		Reference	Reference		Reference
		Occasionally	1.46 (0.94-2.28)		.09	0.57 (0.36-0.91)		.02
		Rarely	1.31 (0.80-2.14)		.28	0.62 (0.37-1.05)		.08
		Never	1.24 (0.74-2.08)		.41	0.64 (0.37-1.11)		.11
	PU		0.79 (0.65-0.97)		.02	1.85 (1.48-2.33)		<.001
	PR		2.09 (1.74-2.51)		<.001	0.77 (0.64-0.92)		.005
**Model^b^**								
	**The frequencies of using GenAI**							
		Mostly	Reference		Reference	Reference		Reference
		Occasionally	1.19 (0.73-1.94)		.48	0.63 (0.37-1.06)		.08
		Rarely	1.02 (0.59-1.78)		.94	0.74 (0.41-1.35)		.33
		Never	1.10 (0.60-2.00)		.76	0.74 (0.39-1.37)		.34
	PU		0.84 (0.66-1.08)		.18	1.56 (1.17-2.08)		.003
	PR		1.80 (1.46-2.21)		<.001	0.86 (0.69-1.08)		.21

^a^Crude models.

^b^Models adjust for age, gender, marital status, ethnicity, salary satisfaction, professional title, years in practice, night shift, weekly patient volume, specialty, teaching assessment pressure, research assessment pressure, and province.

### Associations of GenAI Usage, PU, and PR With Subscales of Burnout

Table 4 shows the results of the fixed-effects linear regression analysis regarding the associations between GenAI factors and two subscales of physician burnout, work exhaustion and interpersonal disengagement. In the crude models and adjusted models, the frequencies of GenAI usage did not show any statistically significant associations with either work exhaustion or interpersonal disengagement compared to the “mostly” user reference group. PU was also not significantly associated with either dimension. However, higher PR was positively associated with both subscales. Specifically, each unit increase in PR was significantly associated with a 0.37-point increase in work exhaustion score (β=0.37, 95% CI 0.30-0.44; *P*<.001) and a 0.34-point increase in interpersonal disengagement score (β=0.34, 95% CI 0.28-0.41; *P*<.001). In the fully adjusted models (refer to footnote b in Table 4), each unit increase in PR was associated with a 0.27-point higher score in both work exhaustion (β=0.27, 95% CI 0.20-0.34; *P*<.001) and interpersonal disengagement (β=0.27, 95% CI 0.19-0.34; *P*<.001).

**Table 4:** Province fixed-effects linear regression analysis of associations of generative AI (GenAI) usage, perceived usefulness (PU), and perceived risk (PR) with subscales of burnout among Chinese physicians.

					Work exhaustion						Interpersonal disengagement			
					β (95% CI)			*P* value			β (95% CI)			*P* value
**Model^a^**														
	**The frequencies of using GenAI**													
		Mostly	Reference			Reference			Reference			Reference		
		Occasionally	0.12 (–0.08 to 0.32)			.24			0.00 (–0.18 to 0.19)			.98		
		Rarely	0.13 (–0.09 to 0.35)			.25			–0.06 (–0.26 to 0.14)			.56		
		Never	0.04 (–0.20 to 0.27)			.75			–0.12 (–0.34 to 0.09)			.25		
	PU			–0.00 (–0.09 to 0.09)			>.99			–0.03 (–0.11 to 0.05)			.50	
	PR			0.37 (0.30 to 0.44)			<.001			0.34 (0.28 to 0.41)			<.001	
**Model^b^**														
	**The frequencies of using GenAI**													
		Mostly	Reference			Reference			Reference			Reference		
		Occasionally	0.05 (–0.14 to 0.24)			.59			–0.09 (–0.27 to 0.10)			.35		
		Rarely	0.08 (–0.13 to 0.29)			.46			–0.16 (–0.36 to 0.04)			.11		
		Never	0.05 (–0.18 to 0.28)			.66			–0.18 (–0.40 to 0.03)			.09		
	PU			0.06 (–0.03 to 0.14)			.21			–0.00 (–0.09 to 0.09)			.97	
	PR			0.27 (0.20 to 0.34)			<.001			0.27 (0.19 to 0.34)			<.001	

^a^Crude models.

^b^Models adjust for age, gender, marital status, ethnicity, salary satisfaction, professional title, years in practice, night shift, weekly patient volume, specialty, teaching assessment pressure, research assessment pressure, and province.

### Subgroup Analysis of Associations of GenAI Use, PU, and PR With Burnout and Fulfillment

The supplementary stratified analyses (Tables S1-S6 in Multimedia Appendix 4) demonstrated that while the main associations between GenAI usage and physician burnout outcomes were largely invariant, the associations involving psychological perceptions exhibited heterogeneities across subgroups. Regarding GenAI usage, its associations with both binary outcomes (burnout and high fulfillment, Table S1 in Multimedia Appendix 4) and continuous burnout subscales (work exhaustion and interpersonal disengagement, Table S2 in Multimedia Appendix 4) remained consistently nonsignificant across almost all strata. A notable exception was a significant interaction for weekly night shifts (*P*_for interaction_=.01); specifically, GenAI usage was uniquely linked to a higher likelihood of burnout among physicians working ≥3 night shifts per week (OR 13.96, 95% CI 2.40-81.04; *P*=.003). For PU, significant interaction effects revealed that its inverse association with burnout was more pronounced among senior physicians with ≥11 years in practice (OR 0.63, 95% CI 0.44-0.91, *P*_for interaction_=.04), and its positive association with high fulfillment was stronger among those with no night shifts or low research pressure (Table S3 in Multimedia Appendix 4). Interestingly, higher PU was associated with a higher interpersonal disengagement specifically among physicians under high research assessment pressure (β=0.13, 95% CI 0.01-0.25; *P*_for interaction_=.03; Table S5 in Multimedia Appendix 4). In addition, the adverse associations of PR were universal; higher PR was independently associated with burnout (Table S4 in Multimedia Appendix 4), work exhaustion, and interpersonal disengagement (Table S6 in Multimedia Appendix 4) across all examined subgroups. Nevertheless, notable heterogeneity was observed across gender, teaching, and research pressure strata. Specifically, the associations of PR with both work exhaustion and interpersonal disengagement were stronger among male physicians than among female physicians (*P*_for interaction_=.03 and *P*_for interaction_<.001, respectively). Moreover, the association between PR and work exhaustion was more pronounced among physicians facing high teaching pressure (*P*_for interaction_=.04), whereas the association between PR and interpersonal disengagement was stronger among those under no or low research assessment pressure (*P*_for interaction_=.048), as shown in Table S6 in Multimedia Appendix 4.

### Qualitative Results

To elucidate the complex associations observed in the quantitative analysis, we conducted thematic analysis on semistructured interviews. Three key themes were identified (Table S1 in Multimedia Appendix 5): (1) explaining the “null” association between GenAI usage and burnout, (2) the positive role of PU in fulfillment, and (3) the detrimental impact of PR on burnout.

#### The “Efficiency-Burden” Paradox: Explaining the Null Association Between GenAI Usage and Burnout

While quantitative data showed no direct correlation between GenAI usage and burnout, qualitative insights revealed a dynamic offsetting effect. GenAI-driven efficiency was often neutralized by an emergent form of invisible labor. Participants reported that while GenAI saved time in data retrieval or drafting, it introduced a “verification tax.” Physicians had to meticulously audit GenAI outputs for accuracy, which creates a cognitive double-shift. As one participant noted:

While GenAI appears to save time, it may essentially be shifting the nature of work rather than reducing it. Unlike other industries, healthcare demands a rigorous and cautious approach. I find myself constantly cross-referencing GenAI outputs with clinical reality, a process that potentially imposes a heavier cognitive load.

Participant 5

Efficiency gains were rarely used for rest but were instead repurposed for higher output.

The system gets faster, but the environment just gets more competitive. We aren’t working less, we are just expected to produce more in the same time.

Participant 10

#### GenAI as a Professional “Multiplier”: PU as a Driver of Professional Fulfillment

The quantitative link between PU and high professional fulfillment was corroborated by the theme of cognitive empowerment. For many, the usefulness of GenAI was synonymous with breaking cognitive boundaries. GenAI was described as a research accelerator and a diagnostic partner for complex cases.

GenAI optimizes my research ideas and streamlines the literature process—this is where I feel most productive. When it helps me solve a difficult diagnostic puzzle, it reinforces my sense of competence.

Participant 4

GenAI provides a more comprehensive range of differential diagnoses and detailed information on drug adverse reactions. Moreover, it inspires new research ideas, truly allowing me to return my focus to clinical care and scientific inquiry.

Participant 6

High PU was linked to the hope that GenAI could take over emotionally draining and repetitive tasks (assistant roles or documentation), allowing physicians to return to the humanistic core of medicine. This shift from clerical worker to decision-maker significantly boosted professional fulfillment.

I am an advocate for GenAI, as I feel I am currently at the most rewarding stage of my career. GenAI significantly alleviates my administrative burden and augments clinical practice, allowing me to return my focus to the humanistic side of clinical care.

Participant 6

#### Erosion of Autonomy and Trust: PR as a Potent Driver of Burnout

PR was identified as a “psychological toxin” that consistently drove burnout by threatening the physician’s professional identity and legal safety. A recurring concern was the legal vulnerability of the physician in the GenAI era.

The GenAI suggests, but I sign. This black box creates a state of hyper-vigilance because the legal accountability is entirely mine, yet the decision logic is partially hidden.

Participant 1

Participants described a deprofessionalization effect where patients used GenAI to challenge physician authority. This tension undermines the physician’s sense of social value, leading to interpersonal disengagement—a core dimension of burnout.

I treat GenAI as a supportive tool rather than a deterministic one. Its recommendations are for reference only. However, when patients bring the prescriptions provided by GenAI to challenge me, it creates a barrier of distrust. It makes the job feel more like a technical battle than a healing profession.

Participant 2

## Discussion

### Principal Findings

Based on a mixed methods approach integrating a large-scale survey and semistructured interviews, this study found that the frequency of GenAI usage was not significantly associated with physician burnout or professional fulfillment. However, the PU of GenAI was correlated with higher levels of professional fulfillment, while PR was significantly associated with a higher risk of burnout. These findings suggest that GenAI adoption per se may not yield direct psychological benefits for clinicians, as the efficiency-driven intensification of workload offsets the anticipated gains of GenAI. Importantly, positive psychological perceptions of GenAI emerged as important correlates of professional fulfillment, while systemic risks and physicians’ negative risk perceptions were key correlates of higher levels of occupational burnout.

### Comparison to Prior Work

Although previous studies have examined the impact of GenAI scribes [14] and diagnostic assistance GenAI [17] on physicians’ burnout, this study yielded distinct results. A critical finding of this study is the null association between the frequency of GenAI usage and physicians’ well-being metrics (burnout and fulfillment). We argue that this efficiency paradox arises because the time-saving benefits of GenAI are systematically offset by two countervailing forces. First, the “efficiency-driven intensification of workload” suggests a manifestation of the Jevons Paradox in digital health care [32]. This paradox holds that although GenAI has enhanced the efficiency of clinical and research tasks, it does not release the capacity of medical staff. The organizational or self-imposed expectations for output volume increase accordingly, thereby generating more unmet needs [33]. From a macro perspective, the application of GenAI in health care has raised service expectations and increased service demand, further straining existing medical resources. At the micro level, this trend leads to a heavier workload for clinicians, offsetting the efficiency gains brought by the technology. As revealed in our qualitative analysis, improved work efficiency has not brought physicians additional rest time. Instead, it has led to a larger patient load and placed health care providers under greater strain. Furthermore, the integration of GenAI introduces a new cognitive cost—a form of invisible labor characterized by the supervision and validation of GenAI outputs. This may merely shift the nature of work rather than reduce the overall workload, similar to the impact of the previous introduction of digital technologies such as electronic health records (EHRs) [34]. This offsets the potential psychological benefits of technological advancement. This may partly explain why the use of GenAI in medical settings presents no clear association with a reduction in physician burnout.

However, we found that physicians’ perceptions of GenAI—whether positive or negative—are intrinsically linked to their psychological well-being. Crucially, the adjusted quantitative analysis demonstrates that PU is positively associated with professional fulfillment but shows no link with burnout-related outcomes, whereas PR emerges as a consistent correlate of occupational burnout. Specifically, within the framework of the JD-R model, PU functions as a critical job resource [21] that bolsters professional fulfillment, though it does not offer a protective buffer against burnout or its subscales. Our quantitative data indicate that higher professional fulfillment is positively associated with physicians’ perceptions regarding GenAI’s capacity to enhance diagnostic accuracy and administrative efficiency. Qualitative interviews further elucidate the psychological mechanism behind this trend, revealing that GenAI does more than merely enhance speed and efficiency; it bolsters physicians’ self-efficacy by providing differential diagnostic support, empowering clinical reasoning, assisting in research design, and alleviating the burden of repetitive documentation [35]. Participants noted that by liberating them from mundane administrative tasks, GenAI allows them to refocus on complex cases, research initiatives, and returning to the bedside—a shift that helps them reclaim their sense of professional value, as corroborated by prior studies [36-39]. This association suggests that when technology is perceived as an augmentative tool that expands professional capabilities, this positive psychological resource can be effectively converted into heightened professional fulfillment [40].

Conversely, our adjusted results show that PR of GenAI is consistently associated with higher levels of burnout. Within the framework of the JD-R model, PR acts as a taxing job demand [21] that is positively associated with burnout-related outcomes, and with no evidence that this association was attenuated by PU. It serves not only as a cognitive stressor—requiring sustained hyper-vigilance—but also as an emotional burden triggered by the potential loss of resources, such as legal penalties and reputational damage [38]. Our quantitative findings demonstrate that physicians’ concerns regarding GenAI-induced clinical harm, service inequality, and patient overreliance are significantly linked to burnout levels. Qualitative interviews further elucidate the mechanism underlying this association: clinicians face a severe accountability-responsibility mismatch. Although GenAI participates in decision-making, the ultimate burden of legal liability and professional reputation remains with the human physician [41]. This accountability black box compels physicians into a chronic state of defensive medical vigilance. Furthermore, this study identified an additional stressor: the challenge GenAI poses to professional authority. When patients use the prescriptions provided by GenAI to question clinical judgments, the resulting social distrust challenges the physician’s professional identity, alienating them from an authoritative healer into a mere algorithm validator. As theorized by Funer and Wiesing, physician autonomy is not an end in itself, but rather finds its justification in its capacity to enhance patient well-being. When this clinical autonomy undergoes a systemic erosion, the mismatch between accountability and liability, coupled with the pressure of cross-referencing, threatens to induce technological deprofessionalization, wherein the clinician’s independent diagnostic agency is compromised [42]. This loss of professional agency not only correlates with elevated work exhaustion but also is tied to interpersonal disengagement—a state in which physicians adopt a cynical and mechanical detachment toward their work [43].

Our main and stratified models reveal an asymmetric dual pathway that deepens the JD-R model in digital health: PU of GenAI as a motivational resource tied to professional fulfillment, whereas PR acts as a taxing job demand linked to burnout and its subscales across almost all subgroups. This asymmetry suggests that tech-driven fulfillment and depletion are independent psychological processes rather than polar opposites. Furthermore, cultivating utility does not shield clinicians from systemic risk-driven stressors. An inversion of this asymmetry was observed under acute systemic strain, most prominently within the highly burdened night-shift subgroup (≥3 days/week). For these exhausted clinicians, GenAI usage was associated with higher burnout risk (OR 13.96). While the wide confidence interval (95% CI 2.40-81.04) indicates limited precision in this subgroup and warrants cautious interpretation regarding the exact magnitude, the lower bound (>2.00) confirms a heightened vulnerability. Stratified analyses unpacked the interaction mechanism behind this risk; within this high-fatigue group, the positive association between PU and professional fulfillment was absent (OR 0.59, *P*_for interaction_=.008), and higher PU was conversely linked with both work exhaustion and interpersonal disengagement. Concurrently, PR showed a strong association with overall burnout (OR 2.79). This points to a potential algorithmic verification burden in the context of fatigue or circadian rhythm disruption. Specifically, exhausted physicians who hold high expectations for GenAI (high PU) are subject to institutional mandates requiring algorithmic cross-checking to avoid clinical harm, a practice that turns the technology into an additional cognitive tax instead of an empowering professional asset. This vulnerability is likewise evidenced by the fact that within groups facing no or lower research pressure, PU was associated with higher fulfillment. Conversely, in the high research pressure group, PU was linked with greater interpersonal disengagement. Future studies may prioritize assessing relevant interventions, including simplifying mandatory algorithmic verification on demanding night shifts, offering personalized support to early-career and high-stress clinicians, and exploring the long-term potential of translating technological benefits into professional fulfillment. The results also have practical implications for hospital technology governance, where differentiated IT strategies and tailored clinician support are worth adopting in daily management.

### Limitations

Despite these insights, several limitations warrant acknowledgment. First, the cross-sectional design precludes the establishment of definitive causal relationships, capturing only the correlations between variables at a single point in time. Consequently, our findings are susceptible to potential reverse causality. For instance, while PR and clinician burnout exhibit a statistical relationship, it is plausible that physicians already experiencing emotional exhaustion adopt a more pessimistic or risk-averse cognitive bias toward adopting new technologies. Second, the study may be subject to selection bias, as physicians with a preexisting interest in GenAI might have been more inclined to participate. Third, the study sample exhibits an imbalance in both geographic and institutional distribution. Geographically, nearly two-thirds of the respondents were clustered in Fujian Province, limiting nationwide generalizability. Institutionally, although the sample included clinicians from 4 distinct regions, participants were predominantly from tertiary hospitals. Consequently, the underrepresentation of primary health care personnel and broader geographic diversity may limit the generalizability of the findings to community-level clinics or diverse cultural contexts. Fourth, the reliance on self-reported data introduces the possibility of social desirability bias [44], which may influence the reporting of sensitive psychological states. Future research should prioritize longitudinal designs to monitor the dynamic evolution of GenAI perceptions, and their long-term associations with burnout or fulfillment. Fifth, the use of subjective, ordinal categories (mostly, occasionally, rarely, and never) to measure GenAI usage frequency introduces potential subjective measurement error. Because these categories lack standardized anchoring (specific hours per week), one participant’s interpretation of “mostly” may differ from another. This subjective variance may introduce underlying noise into our statistical classifications. Sixth, although subgroup analyses yielded novel insights into high-risk populations, certain specific stratifications—such as physicians working ≥3 night shifts per week—yielded small subsample sizes. Consequently, while the strong lower bound confirms a heightened vulnerability, the wide confidence interval reflects reduced statistical precision. Future multicenter studies with larger representative samples are warranted to further refine these subgroup-specific estimations. Finally, our survey measured GenAI usage broadly without recording the specific commercial brands, types of tools used, and specific usage purposes among physicians. Because different platforms vary in their clinical accuracy, user interfaces, cognitive demands, and risk profiles, using a generalized metric may introduce unmeasured heterogeneity into physicians’ perceptions (PU and PR) and potentially mask nuanced differences in psychological impacts.

### Future Directions

To build upon these findings, future research should prioritize longitudinal designs to monitor the temporal evolution of GenAI perceptions and their long-term effects on physician burnout and fulfillment. Additionally, future studies should use more detailed classifications to differentiate the impacts of specific commercial GenAI tools. Comparative investigations across different GenAI modalities—distinguishing between low-risk administrative tools and high-stake clinical decision support systems—would offer more nuanced insights into physician well-being. Finally, the exploration of targeted organizational interventions could provide practical pathways to protect clinician well-being during the ongoing transition to GenAI-integrated health care environments.

### Conclusions

In conclusion, this study evaluated the relationships between GenAI usage, physicians’ psychological perceptions (PU and PR), and clinician well-being (burnout and professional fulfillment). Quantitative and qualitative data revealed that while PU was positively linked to professional fulfillment, PR was a prominent correlate of clinician burnout, reflecting legal vulnerability and identity threats. Notably, the null association between GenAI usage and burnout underscored an efficiency-burden paradox, where administrative time-savings were offset by the invisible cognitive load of output verification. Sustaining clinician well-being during this digital shift thus requires a dual requirement, balancing the potential for professional fulfillment tied to GenAI utility against the concurrent verification fatigue and legal uncertainty clustering around clinician burnout.

## Appendix

Multimedia Appendix 1 Sample selection, regional economic characteristics, and sample distribution.

Multimedia Appendix 2 Survey items and scale reliability and validity.

Multimedia Appendix 3 Interviewees' characteristics and theme saturation matrix.

Multimedia Appendix 4 Subgroup analysis.

Multimedia Appendix 5 Qualitative interpretation.

## Abbreviations

AVE: average variance extracted
CFA: confirmatory factor analysis
CFI: comparative fit index
CMV: common method variance
CR: composite reliability
CV: convergent validity
DAT: diagnostic and analytical type
DMT: decision-making type
EFA: exploratory factor analysis
EHR: electronic health record
GDP: gross domestic product
GenAI: generative AI
JD-R: job demands-resources
OR: odds ratio
PFI: professional fulfillment index
POT: procedural and operative type
PR: perceived risk
PU: perceived usefulness
RCT: relational and communicative type
RMSEA: root-mean-square error of approximation
SRMR: standardized root-mean-square residual
TAM: technology acceptance model
TLI: Tucker-Lewis Index
VIF: variance inflation factor

## Data Availability

The corresponding author had full access to all data in the study and ﬁnal responsibility for the decision to submit the report for publication. The data used and analyzed during the current study are available from the corresponding author upon reasonable request.
