# Supplementary material for: Generative AI Use, Perceived Usefulness, Perceived Risk, and Physician Burnout and Fulfillment Among Chinese Physicians: Mixed Methods Multiregional Study
Source: J Med Internet Res. 2026 Sep 11;28:e94155. doi: 10.2196/94155 (PMC13615376; doi:10.2196/94155)
Supplement: Supplementary file 1 [file jmir_v28i1e94155_app1.docx]

**Multimedia Appendix 1**

**Sample selection, regional economic characteristics, and sample distribution**

**Figure S1.** Flow diagram illustrating the participant recruitment, exclusion, and final analytical sample selection process for the cross-sectional survey.

**Table S1**. Regional economic characteristics and sample distribution across multiple provinces in China.

| Region | City/District | Sample size  (n) | Percentage  (%) | GDP per Capita  (¥, 2023) | Economic level |
| --- | --- | --- | --- | --- | --- |
| Fujian | Ningde | 619 | 64.4 | 120,619 | High |
| Sichuan | Luzhou | 127 | 13.2 | 63,913 | Moderate |
| Beijing | Fengtai | 118 | 12.3 | 200,278 | Very high |
| Shanxi | Taiyuan | 97 | 10.1 | 102,922ᵃ | Moderate-high |
| Total |  | 961 | 100.0 |  |  |

ᵃ Most recent available data (2022).

Economic development data were obtained from the National Bureau of Statistics of China and respective municipal statistical bureaus.

Economic level categorization: Very High (GDP per capita > 180% of national average ¥89,358, in 2023), High (120-180%), Moderate-High (100-120%), Moderate (60-100%).
