# Supplementary material for: Generative AI Use, Perceived Usefulness, Perceived Risk, and Physician Burnout and Fulfillment Among Chinese Physicians: Mixed Methods Multiregional Study
Source: J Med Internet Res. 2026 Sep 11;28:e94155. doi: 10.2196/94155 (PMC13615376; doi:10.2196/94155)
Supplement: Supplementary file 2 [file jmir_v28i1e94155_app2.docx]

**Multimedia Appendix 2**

**Survey items and scale reliability and validity**

**Table S1.** Survey items of perceived usefulness (PU) and perceived risk (PR) of generative artificial intelligence (GenAI).

| Item | Item wording |
| --- | --- |
| PU |  |
| PU1 | GenAI improves/has improved the accuracy of diagnosis. |
| PU2 | GenAI improves/has improved the efficiency of documentation. |
| PU3 | GenAI improves/has improved overall work efficiency. |
| PR |  |
| PR1 | GenAI may increase the risk of patient harm. |
| PR2 | GenAI may increase the risk of inequity in healthcare delivery. |
| PR3 | GenAI may lead to patient over-reliance on technology rather than seeking professional medical help. |

**Table S2.** Reliability and validity analysis of the measurement scales for perceived generative artificial intelligence (GenAI) characteristics among Chinese physicians.

| Item | Standardized loading | Cronbach’s α | CR | AVE |
| --- | --- | --- | --- | --- |
| Perceived usefulness (PU) |  | 0.81 | 0.81 | 0.59 |
| PU1 | 0.79 |  |  |  |
| PU2 | 0.72 |  |  |  |
| PU3 | 0.80 |  |  |  |
| Perceived risk (PR) |  | 0.82 | 0.84 | 0.63 |
| PR1 | 0.87 |  |  |  |
| PR2 | 0.87 |  |  |  |
| PR3 | 0.62 |  |  |  |

**CV: c**onvergent validity; AVE:average variance extracted.

**Table S3.** Discriminant validity results for perceived generative artificial intelligence (GenAI) via the Fornell-Larcker criterion.

| Variable | 1. Perceived usefulness | 2. Perceived risk |
| --- | --- | --- |
| 1. Perceived usefulness (PU) | 0.767 |  |
| 2. Perceived risk (PR) | -0.030 | 0.795 |

Note: Values on the diagonal represent the square root of the average variance extracted (AVE) for each construct, while the off-diagonal value represents the correlation coefficient between the constructs.
