# Supplementary material for: Generative AI Use, Perceived Usefulness, Perceived Risk, and Physician Burnout and Fulfillment Among Chinese Physicians: Mixed Methods Multiregional Study
Source: J Med Internet Res. 2026 Sep 11;28:e94155. doi: 10.2196/94155 (PMC13615376; doi:10.2196/94155)
Supplement: Supplementary file 3 [file jmir_v28i1e94155_app3.docx]

**Multimedia Appendix 3**

**Interviewees' characteristics and theme saturation matrix**

**Table S1.** The basic characteristics of the interviewees in the qualitative study (N=10).

| ID | Gender | Age | Specialty | Years in practice | Burnout level |
| --- | --- | --- | --- | --- | --- |
| P01 | Male | 40 | POT | 13 | Severe |
| P02 | Female | 31 | RCT | 1.5 | Moderate |
| P03 | Female | 28 | DMT | 3 | Mild or no |
| P04 | Female | 47 | DMT | 20 | Mild or no |
| P05 | Female | 26 | DAT | 1.5 | Mild or no |
| P06 | Female | 37 | POT | 13 | Moderate |
| P07 | Female | 50 | DMT | 30 | Moderate |
| P08 | Male | 36 | POT | 10 | Mild or no |
| P09 | Male | 53 | RCT | 25 | Moderate |
| P10 | Male | 26 | DAT | 1 | Severe |

DAT, diagnostic and analytical type; POT, procedural and operative type; DMT, decision-making type; RCT, relational and communicative type.

**Table S2.** Thematic saturation matrix for the qualitative study.

| Main theme | Subthemes | P01 | P02 | P03 | P04 | P05 | P06 | P07 | P08 | P09 | P10 |
| --- | --- | --- | --- | --- | --- | --- | --- | --- | --- | --- | --- |
| 1. The “efficiency-burden” paradox | Verification tax & cognitive double-shift | ✓ |  | ✓ |  | ✓ | ✓ | ✓ |  | ✓ |  |
|  | Repurposed efficiency |  | ✓ |  | ✓ |  |  | ✓ |  | ✓ | ✓ |
| 2. GenAI as a professional “multiplier” | Cognitive empowerment |  | ✓ | ✓ | ✓ |  | ✓ | ✓ | ✓ | ✓ | ✓ |
|  | Reclaiming the humanistic core |  |  |  |  | ✓ | ✓ |  |  | ✓ |  |
| 3. Erosion of autonomy and trust | The accountability black box | ✓ |  | ✓ | ✓ | ✓ |  | ✓ | ✓ | ✓ | ✓ |
|  | De-professionalization & trust erosion |  | ✓ |  |  | ✓ | ✓ |  | ✓ | ✓ |  |
| New theme emerged? |  | Yes | Yes | No | No | Yes | No | No | No | No | No |
