# Supplementary material for: Generative AI Use, Perceived Usefulness, Perceived Risk, and Physician Burnout and Fulfillment Among Chinese Physicians: Mixed Methods Multiregional Study
Source: J Med Internet Res. 2026 Sep 11;28:e94155. doi: 10.2196/94155 (PMC13615376; doi:10.2196/94155)
Supplement: Supplementary file 4 [file jmir_v28i1e94155_app4.docx]

**Multimedia Appendix 4**

**Subgroup analysis**

**Table S1.** Subgroup analyses of the associations of generative artificial intelligence (GenAI) usage frequency with physician burnout and high professional fulfillment.

| Characteristics | Burnout | |  | High fulfillment | |
| --- | --- | --- | --- | --- | --- |
|  | OR (95% CI) | *P _for interaction_* |  | OR (95% CI) | *P _for interaction_* |
| Age |  |  |  |  |  |
| ≤35 | 1.06 (0.60,1.90) | .60 |  | 1.03 (0.55,1.92) | .84 |
| ≥36 | 0.97 (0.48,1.94) |  |  | 0.91 (0.48,1.74) |  |
| Gender |  |  |  |  |  |
| Male | 0.77 (0.37,1.62) | .52 |  | 1.05 (0.52,2.12) | .73 |
| Female | 1.12 (0.63,1.98) |  |  | 1.04 (0.56,1.92) |  |
| Years in practice |  |  |  |  |  |
| 1-10 | 0.98 (0.54,1.78) | .93 |  | 1.13 (0.59,2.13) | .57 |
| ≥11 | 1.23 (0.62,2.45) |  |  | 0.79 (0.43,1.48) |  |
| Night shift per week |  |  |  |  |  |
| ≥3 days | 13.96 (2.40,81.04)^**^ | .01 |  | 0.37 (0.10,1.36) | .07 |
| 1-2 days | 0.80 (0.44,1.47) |  |  | 0.86 (0.46,1.59) |  |
| No | 0.68 (0.27,1.69) |  |  | 1.62 (0.65,4.06) |  |
| Specialty |  |  |  |  |  |
| DAT | 1.56 (0.47,5.22) | .88 |  | 3.24 (0.66,15.94) | .06 |
| POT | 0.57 (0.23,1.40) |  |  | 1.37 (0.61,3.07) |  |
| DMT | 1.88 (0.88,4.00) |  |  | 0.69 (0.34,1.44) |  |
| RCT | 0.42 (0.11,1.57) |  |  | 0.32 (0.08,1.19) |  |
| Teaching assessment pressure | | | | | |
| No or low-pressure | 1.01 (0.63,1.63) | .75 |  | 1.02 (0.60,1.74) | .69 |
| High-pressure | 0.75 (0.20,2.83) |  |  | 1.07 (0.47,2.43) |  |
| Research assessment pressure | | | | | |
| No or low-pressure | 1.21 (0.73,2.02) | .30 |  | 0.80 (0.47,1.38) | .30 |
| High-pressure | 0.64 (0.25,1.65) |  |  | 1.79 (0.75,4.25) |  |

DAT, diagnostic and analytical type; POT, procedural and operative type; DMT, decision-making type; RCT, relational and communicative type.

All the models adjust for age, gender, marital status, ethnicity, salary satisfaction, professional title, years in practice, night shift, weekly patient volume, specialty, teaching assessment pressure, research assessment pressure, province except for the stratification variables.

*^**^P< .01.*

**Table S2.** Subgroup analyses of the associations of generative artificial intelligence (GenAI) usage frequency with the burnout subscales.

| Characteristics | Work exhaustion | |  | Interpersonal disengagement | |
| --- | --- | --- | --- | --- | --- |
|  | β (95% CI) | *P _for interaction_* |  | β (95% CI) | *P _for interaction_* |
| Age |  |  |  |  |  |
| ≤35 | -0.03 (-0.22,0.17) | .82 |  | 0.14(-0.05,0.33) | .24 |
| ≥36 | 0.04 (-0.17,0.25) |  |  | -0.01(-0.20,0.18) |  |
| Gender |  |  |  |  |  |
| Male | -0.12 (-0.32,0.09) | .10 |  | -0.01(-0.20,0.18) | .09 |
| Female | 0.13 (-0.07,0.33) |  |  | 0.18(-0.01, 0.37) |  |
| Years in practice |  |  |  |  |  |
| 1-10 | 0.02 (-0.18,0.22) | .85 |  | 0.11 (-0.09, 0.30) | .65 |
| ≥11 | 0.05 (-0.16,0.25) |  |  | 0.11 (-0.08, 0.29) |  |
| Night shift per week |  |  |  |  |  |
| ≥3 days | 0.09 (-0.33,0.51) | .51 |  | 0.35 (-0.09, 0.79) | .05 |
| 1-2 days | -0.01 (-0.19,0.17) |  |  | 0.10 (-0.07, 0.26) |  |
| No | -0.02 (-0.33,0.29) |  |  | -0.08 (-0.36, 0.19) |  |
| Specialty |  |  |  |  |  |
| DAT | 0.12 (-0.29,0.53) | .78 |  | 0.16 (-0.23, 0.55) | .72 |
| POT | -0.09 (-0.36,0.17) |  |  | -0.01 (-0.26, 0.24) |  |
| DMT | 0.05 (-0.16,0.26) |  |  | 0.20 (-0.01, 0.40) |  |
| RCT | 0.14 (-0.33,0.60) |  |  | 0.06 (-0.34, 0.46) |  |
| Teaching assessment pressure | | | | | |
| No or low-pressure | 0.03 (-0.13,0.19) | .28 |  | 0.10 (-0.05, 0.25) | .56 |
| High-pressure | -0.12 (-0.41,0.18) |  |  | -0.01 (-0.31, 0.29) |  |
| Research assessment pressure | | | | | |
| No or low-pressure | 0.02 (-0.14,0.19) | .58 |  | 0.09 (-0.06, 0.25) | .83 |
| High-pressure | -0.06 (-0.33,0.22) |  |  | 0.08 (-0.17, 0.33) |  |

DAT, diagnostic and analytical type; POT, procedural and operative type; DMT, decision-making type; RCT, relational and communicative type.

All the models adjust for age, gender, marital status, ethnicity, salary satisfaction, professional title, years in practice, night shift, weekly patient volume, specialty, teaching assessment pressure, research assessment pressure, province except for the stratification variables.

**Table S3.** Subgroup analyses of the associations of perceived usefulness (PU) with physician burnout and high professional fulfillment.

| Characteristics | Burnout | |  | High fulfillment | |
| --- | --- | --- | --- | --- | --- |
|  | OR (95% CI) | *P _for_ _interaction_* |  | OR (95% CI) | *P _for interaction_* |
| Age |  |  |  |  |  |
| ≤35 | 1.07 (0.73, 1.57) | .09 |  | 1.12 (0.71, 1.77) | .09 |
| ≥36 | 0.68 (0.48, 0.98)^*^ |  |  | 1.97 (1.35, 2.90)^**^ |  |
| Gender |  |  |  |  |  |
| Male | 0.79 (0.55, 1.13) | .87 |  | 2.02 (1.37, 2.96)^***^ | .11 |
| Female | 0.80 (0.56, 1.15) |  |  | 1.20 (0.78, 1.85) |  |
| Years in practice |  |  |  |  |  |
| 1-10 | 1.12 (0.77, 1.62) | .04 |  | 1.19 (0.75, 1.89) | .16 |
| ≥11 | 0.63 (0.44, 0.91)**^*^** |  |  | 1.92 (1.31, 2.81)**^**^** |  |
| Night shift per week |  |  |  |  |  |
| ≥3 days | 1.44 (0.57, 3.63) | .10 |  | 0.59 (0.27, 1.31) | .008 |
| 1-2 days | 0.85 (0.63, 1.17) |  |  | 1.65 (1.12, 2.42)^*^ |  |
| No | 0.52 (0.30, 0.89)**^*^** |  |  | 2.43 (1.38, 4.29)^**^ |  |
| Specialty |  |  |  |  |  |
| DAT | 0.69 (0.29, 1.62) | .85 |  | 3.93 (1.39,11.18)^*^ | .24 |
| POT | 0.91 (0.60, 1.39) |  |  | 1.65 (1.05, 2.60)^*^ |  |
| DMT | 1.07 (0.69, 1.65) |  |  | 1.31 (0.81, 2.11) |  |
| RCT | 0.52 (0.24, 1.14) |  |  | 1.54 (0.62, 3.82) |  |
| Teaching assessment pressure | | | | | |
| No or low-pressure | 0.88 (0.66, 1.18) | .42 |  | 1.58 (1.09, 2.29)^*^ | .71 |
| High-pressure | 0.75 (0.45, 1.25) |  |  | 1.57 (0.99, 2.50) |  |
| Research assessment pressure | |  |  |  |  |
| No or low-pressure | 0.67 (0.49, 0.92)^*^ | .06 |  | 2.05 (1.35, 3.12)^**^ | .04 |
| High-pressure | 1.40 (0.88, 2.25) |  |  | 1.13 (0.74, 1.75) |  |

DAT, diagnostic and analytical type; POT, procedural and operative type; DMT, decision-making type; RCT, relational and communicative type.

All the models adjust for age, gender, marital status, ethnicity, salary satisfaction, professional title, years in practice, night shift, weekly patient volume, specialty, teaching assessment pressure, research assessment pressure, province except for the stratification variables.

*^*^P< .05, ^**^P< .01, ^***^P< .001.*

**Table S4.** Subgroup analyses of the associations of perceived risk (PR) with physician burnout and high professional fulfillment.

| Characteristics | Burnout | |  | High fulfillment | |
| --- | --- | --- | --- | --- | --- |
|  | OR (95% CI) | *P _for interaction_* |  | OR (95% CI) | *P _for interaction_* |
| Age |  |  |  |  |  |
| ≤35 | 1.92 (1.46, 2.53)^***^ | .63 |  | 0.78 (0.56, 1.09) | .09 |
| ≥36 | 1.65 (1.19, 2.28)^**^ |  |  | 0.91 (0.66, 1.24) |  |
| Gender |  |  |  |  |  |
| Male | 2.21 (1.58, 3.11)^***^ | .09 |  | 0.96 (0.69, 1.34) | .27 |
| Female | 1.58 (1.19, 2.10)^**^ |  |  | 0.71 (0.52, 0.98)^*^ |  |
| Years in practice |  |  |  |  |  |
| 1-10 | 1.85 (1.40, 2.45)^***^ | .89 |  | 0.81 (0.58, 1.13) | .88 |
| ≥11 | 1.73 (1.25, 2.39)^**^ |  |  | 0.88 (0.64, 1.20) |  |
| Night shift per week |  |  |  |  |  |
| ≥3 days | 2.79 (1.18, 6.62)^*^ | .51 |  | 0.63 (0.31, 1.27) | .65 |
| 1-2 days | 1.74 (1.34, 2.25)^***^ |  |  | 0.85 (0.63, 1.14) |  |
| No | 2.06 (1.26, 3.35)^**^ |  |  | 0.80 (0.48, 1.34) |  |
| Specialty |  |  |  |  |  |
| DAT | 1.84 (0.90, 3.76) | .65 |  | 0.60 (0.28, 1.31) | .88 |
| POT | 1.67 (1.18, 2.37)^**^ |  |  | 1.04 (0.71, 1.52) |  |
| DMT | 2.22 (1.54, 3.19)^***^ |  |  | 0.79 (0.54, 1.14) |  |
| RCT | 1.35 (0.76, 2.39) |  |  | 0.70 (0.41, 1.19) |  |
| Teaching assessment pressure | | | | | |
| No or low-pressure | 1.74 (1.37, 2.19)^***^ | .31 |  | 0.77 (0.59, 1.02) | .17 |
| High-pressure | 2.38 (1.49, 3.78)^***^ |  |  | 1.00 (0.66, 1.52) |  |
| Research assessment pressure | | | | | |
| No or low-pressure | 1.85 (1.42, 2.40)^***^ | .54 |  | 0.76 (0.55, 1.06) | .13 |
| High-pressure | 1.71 (1.19, 2.46)^**^ |  |  | 1.00 (0.71, 1.42) |  |

DAT, diagnostic and analytical type; POT, procedural and operative type; DMT, decision-making type; RCT, relational and communicative type.

All the models adjust for age, gender, marital status, ethnicity, salary satisfaction, professional title, years in practice, night shift, weekly patient volume, specialty, teaching assessment pressure, research assessment pressure, province except for the stratification variables.

*^*^P< .05, ^**^P< .01, ^***^P< .001.*

**Table S5.** Subgroup analyses of the associations of perceived usefulness (PU) with the burnout subscales.

| Characteristics | Work exhaustion | |  | Interpersonal disengagement | |
| --- | --- | --- | --- | --- | --- |
|  | β (95% CI) | *P _for interaction_* |  | β (95% CI) | *P _for interaction_* |
| Age |  |  |  |  |  |
| ≤35 | 0.12 (0.00, 0.24)^*^ | .26 |  | 0.09 (-0.03, 0.20) | .03 |
| ≥36 | 0.01 (-0.09, 0.12) |  |  | -0.06 (-0.15, 0.03) |  |
| Gender |  |  |  |  |  |
| Male | 0.07 (-0.03, 0.18) | .48 |  | -0.01 (-0.11, 0.09) | .60 |
| Female | -0.00 (-0.12, 0.11) |  |  | -0.01 (-0.12, 0.10) |  |
| Years in practice |  |  |  |  |  |
| 1-10 | 0.12 (0.00, 0.24)^*^ | .13 |  | 0.10 (-0.02, 0.22) | .007 |
| ≥11 | -0.00 (-0.10, 0.10) |  |  | -0.08 (-0.17, 0.01) |  |
| Night shift per week |  |  |  |  |  |
| ≥3 days | 0.34 (0.11, 0.58)^**^ | .16 |  | 0.26 (0.02, 0.51)^*^ | .07 |
| 1-2 days | 0.02 (-0.08, 0.11) |  |  | -0.00 (-0.09, 0.09) |  |
| No | 0.03 (-0.16, 0.22) |  |  | -0.09 (-0.26, 0.08) |  |
| Specialty |  |  |  |  |  |
| DAT | 0.08 (-0.16, 0.32) | .77 |  | -0.06 (-0.29, 0.17) | .71 |
| POT | 0.03 (-0.11, 0.17) |  |  | -0.00 (-0.14, 0.13) |  |
| DMT | 0.10 (-0.02, 0.21) |  |  | 0.07 (-0.04, 0.18) |  |
| RCT | -0.09 (-0.40, 0.21) |  |  | -0.18 (-0.45, 0.08) |  |
| Teaching assessment pressure | | | | | |
| No or low-pressure | 0.04 (-0.05, 0.14) | .92 |  | 0.02 (-0.07, 0.11) | .32 |
| High-pressure | 0.05 (-0.08, 0.18) |  |  | -0.04 (-0.17, 0.09) |  |
| Research assessment pressure | | | | | |
| No or low-pressure | 0.01 (-0.09, 0.11) | .38 |  | -0.07 (-0.16, 0.03) | .03 |
| High-pressure | 0.11 (-0.02, 0.24) |  |  | 0.13 (0.01, 0.25)^*^ |  |

DAT, diagnostic and analytical type; POT, procedural and operative type; DMT, decision-making type; RCT, relational and communicative type.

All the models adjust for age, gender, marital status, ethnicity, salary satisfaction, professional title, years in practice, night shift, weekly patient volume, specialty, teaching assessment pressure, research assessment pressure, province except for the stratification variables.

*^*^P< .05, ^**^P< .01.*

**Table S6.** Subgroup analyses of the associations of perceived risk (PR) with the burnout subscales.

| Characteristics | Work exhaustion | |  | Interpersonal disengagement | |
| --- | --- | --- | --- | --- | --- |
|  | β (95% CI) | *P _for interaction_* |  | β (95% CI) | *P _for interaction_* |
| Age |  |  |  |  |  |
| ≤35 | 0.30 (0.21, 0.39)^***^ | .36 |  | 0.30 (0.21, 0.39)^***^ | .19 |
| ≥36 | 0.22 (0.12, 0.32)^***^ |  |  | 0.21 (0.12, 0.30)^***^ |  |
| Gender |  |  |  |  |  |
| Male | 0.34 (0.24, 0.43)^***^ | .03 |  | 0.39 (0.30, 0.48)^***^ | <.001 |
| Female | 0.20 (0.11, 0.29)^***^ |  |  | 0.14 (0.06, 0.23)^**^ |  |
| Years in practice |  |  |  |  |  |
| 1-10 | 0.28 (0.19, 0.37)^***^ | .79 |  | 0.30 (0.21, 0.38)^***^ | .26 |
| ≥11 | 0.25 (0.15, 0.34)^***^ |  |  | 0.21 (0.13, 0.30)^***^ |  |
| Night shift per week |  |  |  |  |  |
| ≥3 days | 0.30 (0.09, 0.51)^**^ | .57 |  | 0.30 (0.08, 0.51)^**^ | .97 |
| 1-2 days | 0.25 (0.17, 0.33)^***^ |  |  | 0.25 (0.18, 0.32)^***^ |  |
| No | 0.35 (0.17, 0.52)^***^ |  |  | 0.25 (0.09, 0.40)^**^ |  |
| Specialty |  |  |  |  |  |
| DAT | 0.25 (0.04, 0.46)^*^ | .07 |  | 0.28 (0.08, 0.48)^**^ | .07 |
| POT | 0.32 (0.20, 0.43)^***^ |  |  | 0.29 (0.18, 0.40)^***^ |  |
| DMT | 0.28 (0.17, 0.38)^***^ |  |  | 0.26 (0.16, 0.36)^***^ |  |
| RCT | 0.16 (-0.04, 0.36) |  |  | 0.22 (0.06, 0.39)^*^ |  |
| Teaching assessment pressure | | | | | |
| No or low-pressure | 0.23 (0.16, 0.31)^***^ | .04 |  | 0.24 (0.17, 0.31)^***^ | .12 |
| High-pressure | 0.42 (0.29, 0.55)^***^ |  |  | 0.36 (0.23, 0.49)^***^ |  |
| Research assessment pressure | | | | | |
| No or low-pressure | 0.29 (0.20, 0.37)^***^ | .24 |  | 0.29 (0.22, 0.37)^***^ | .048 |
| High-pressure | 0.22 (0.11, 0.34)^***^ |  |  | 0.18 (0.08, 0.29)^***^ |  |

DAT, diagnostic and analytical type; POT, procedural and operative type; DMT, decision-making type; RCT, relational and communicative type.

All the models adjust for age, gender, marital status, ethnicity, salary satisfaction, professional title, years in practice, night shift, weekly patient volume, specialty, teaching assessment pressure, research assessment pressure, province except for the stratification variables.

*^*^P< .05, ^**^P< .01, ^***^P< .001.*
