# Supplementary material for: Generative AI Use, Perceived Usefulness, Perceived Risk, and Physician Burnout and Fulfillment Among Chinese Physicians: Mixed Methods Multiregional Study
Source: J Med Internet Res. 2026 Sep 11;28:e94155. doi: 10.2196/94155 (PMC13615376; doi:10.2196/94155)
Supplement: Supplementary file 5 [file jmir_v28i1e94155_app5.docx]

**Multimedia Appendix 5**

**Qualitative interpretation**

**Table S1.** Qualitative explanations for quantitative findings based on a sequential explanatory design.

| Main theme | Subthemes | Explanatory link to quantitative results | Representative quotes |
| --- | --- | --- | --- |
| 1. The “efficiency-burden” paradox | Verification tax & cognitive double-shift | Explaining the null association: The time saved by GenAI is neutralized by invisible labor (auditing outputs), preventing a net reduction in total workload. | “*While* *GenAI appears to save time, it may essentially be shifting the nature of work... I find myself constantly cross-referencing GenAI outputs with clinical reality, a process that potentially imposes a heavier cognitive load*.” (P05) |
|  | Repurposed efficiency | Efficiency gains are absorbed by systemic demands for higher output rather than being converted into rest. | “*The system gets faster, but the environment just gets more competitive. We aren’t working less, we are just expected to produce more in the same time.*” (P10) |
| 2. GenAI as a professional “multiplier” | Cognitive empowerment | Explaining the positive link to fulfillment: GenAI acts as a diagnostic partner and research accelerator, enhancing physicians' sense of competence and mastery. | “*When it (GenAI) helps me solve a difficult diagnostic puzzle, it reinforces my sense of competence.”* (P04)  *“...truly allowing me to return my focus to clinical care and scientific inquiry.*” (P06) |
|  | Reclaiming the humanistic core | High PU is linked to the expectation that GenAI will absorb clerical drudgery, allowing a return to the role of a professional decision-maker. | “*GenAI significantly alleviates my administrative burden and augments clinical practice, allowing me to return my focus to the humanistic side of clinical care.*” (P06) |
| 3. Erosion of autonomy and trust | The accountability black box | Explaining PR as a burnout predictor: The asymmetry of responsibility (GenAI suggests, physician signs) creates a state of chronic hyper-vigilance. | “*The GenAI suggests, but I sign. This black box creates a state of hyper-vigilance because the legal accountability is entirely mine, yet the decision logic is partially hidden.*” (P01) |
|  | De-professionalization & trust erosion | Risk perceptions directly fuel depersonalization (a core burnout dimension) through physician-patient conflict and perceived loss of social value. | “*When patients bring the prescriptions provided by GenAI to challenge me, it creates a barrier of distrust. It makes the job feel more like a technical battle than a healing profession.*” (P02) |
